# Difference in Response to Feedback and Gender in Three Therapeutic Community Units

**DOI:** 10.3389/fpsyt.2021.690713

**Published:** 2021-06-30

**Authors:** Keith Warren, Nathan J. Doogan, Fiona Doherty

**Affiliations:** ^1^Ohio Colleges of Medicine Government Resource Center, The Ohio State University, Columbus, OH, United States; ^2^Government Resource Center, College of Medicine, The Ohio State University, Columbus, OH, United States

**Keywords:** therapeutic community, gender, substance abuse and addiction, substance abuse treatment, mutual aid, social network analysis

## Abstract

Therapeutic communities (TCs) for substance abuse incorporate a system of peer feedback through written affirmations and corrections. Previous research has found that TC residents show a response to affirmations that is detectable for roughly 8 weeks, with response to corrections being of shorter duration and weaker overall. It is not clear whether and to what extent response to feedback in TCs varies between men and women. Previous research in other settings suggests that women should be more responsive to feedback than men. In order to test this hypothesis we draw on a large dataset of affirmations and corrections sent and received in three 80 bed TC units, two of which house men and one of which houses women. The analysis uses a multilevel negative binomial model, treating affirmations and corrections that TC residents receive as predictors of affirmations that they send over a 9 week period (week 0, the week during which affirmations and corrections are actually sent, and eight subsequent weeks). The model controls for gender, age, race, unit and scores on the Level of Service Inventory-Revised (LSI-R). The relationship between affirmations received and those sent is stronger for women during the initial week and on lags 1-2 and 5-8. The relationship between corrections received and affirmations sent is stronger for women on lags 2 and 8. Graphs suggest that response to affirmations falls off in an exponential curve, while that to corrections appears to include a periodic element. These results indicate that both men and women respond to feedback, but that the strength of the women's response is somewhat greater. These results suggest that any difference in suitability by gender to the feedback approach that characterizes TCs may favor women.

## Introduction

Therapeutic communities (TCs) are residential programs for substance abuse recovery in which mutual aid within the community of peers forms the core approach to treatment (1, 2). TCs work to bring about resident change through a combination of clear behavioral expectations, work at jobs that are necessary for the functioning of the unit, the use of staff and senior peers as role models and feedback between residents (1). Several systematic reviews and meta-analyses have found that TCs are effective in reducing substance abuse and the likelihood of re-incarceration (3–8), although agreement is not universal (9), and there is evidence that effects decline over time, suggesting the importance of aftercare (10).

The extent to which TCs are appropriate or effective for women has also been questioned. There is evidence that women have a more communal attitude than men (11) and that women hold a more interdependent sense of self, considering their relationship with peers as being more symbiotic (12). The repeated finding that women are more active in their social networks than men also suggests that women place a high value on relationships (13, 14). Given this evidence, it is not surprising that that both peer and staff interactions are significant for retention among women in substance abuse treatment (15). However, qualitative research has found that women may have difficulty in forming meaningful relationships with peers in TCs (16), even when the TCs are gender segregated (17). Difficulty in forming peer relationships appears to be a challenge in other correctional settings, with women sometimes reporting that it is easier to form relationships with staff than with peers (18). But this issue is likely to be particularly salient for TCs, given the programs' emphasis on the entire community of staff and peers as the method of treatment (1, 2).

Outcome findings of studies of TC treatment with women have been equivocal. One randomized controlled trial conducted with 115 women found that gender responsive treatment, a manualized combination of group therapy and individual counseling, led to greater reductions in drug abuse, longer aftercare treatment and lower rates of re-incarceration than TC treatment (19). However, a randomized trial comparing gender-sensitive TC treatment to gender-sensitive cognitive behavioral therapy treatment found that TC treatment was more effective in reducing substance abuse, criminal behavior and exposure to trauma, as well as improving mental health scores (20). This would suggest that, while gender sensitivity is an issue in TC treatment, the actual modality itself may not be problematic for women; this would be consistent with meta-analytic results of the broader area of gender sensitive treatment for female offenders (21). In addition, one randomized controlled trial of unmodified TC treatment vs. cognitive behavioral therapy with female prisoners found that TC treatment led to better mental health and criminal behavior outcomes (22). A recent systematic review of treatment for female criminal offenders found little evidence in favor of any treatment (23), while another suggested that some elements of TC treatment could be beneficial in working with women offenders (24).

In light of the contradictory and somewhat complex corpus of studies on women and TCs, it is worth noting that there is a body of theory and empirical results which support the idea that women should have outcomes in TCs that are equal or superior to those of men. The ability to cooperate with peers is a critical aspect of TC treatment (25, 26). Studies suggest that women have advantages in cooperative behavior. They are more empathetic than men (27), show greater altruism (28, 29), and are more likely to resolve conflict harmoniously (30). Researchers have found women to be more cooperative in public goods games, in which members of a team choose to make or not make individual sacrifices that will benefit the group, although contextual variables can influence this (31). There is experimental evidence that women prefer to cooperate with other women and are more likely to pay money to punish peers who defect; the authors took this as indicating that women value the social interactions involved in the game over any profit to be made (32).

Moreover, there is experimental evidence that women are more likely to allow feedback to influence their behavior (33), apparently because they are more likely than men to think that the information is of value (34). This suggests that women should respond more strongly than men to the TC system of ongoing feedback between peers, which in turn is an important source of social learning in the programs (1, 2, 25).

Peer feedback in TCs comes in a variety of forms, including supervision of junior residents in work tasks, frank exchanges during group therapy, affirmations for pro-social behavior such as talking with a peer who is having a difficult time in the program and corrections for behavior that contravenes TC norms, such as demeaning a peer or even doing a poor job on a chore. While most of these forms of peer feedback go unrecorded, TCs sometimes keep written records of peer affirmations and corrections for purposes of monitoring clinical progress.

In units where such records are kept it is possible to measure resident response to peer feedback using longitudinal social network analysis (35). Since peer affirmations themselves are a form of pro-social behavior, one can treat the affirmations and corrections that TC residents receive during one time period as a predictor of the affirmations they send in later time periods. Any increase or decrease in the number of affirmations residents send following the reception of an affirmation or correction forms a measure of resident response to peer feedback.

Previous research using this method did not find any gender difference in the number of affirmations that TC residents sent (35). However, this only tells us that women and men participate in the peer feedback system at roughly similar levels. It does not tell us whether women respond differently to peer feedback than men. Since women are often more cooperative than men and appear to value social relations more highly (11, 31, 32), and since experimental evidence suggests that they are more responsive to feedback (33, 34) we would expect them to be more responsive to the peer feedback system in TCs. If so, clinicians and researchers should be somewhat cautious in altering this system when modifying TCs for women. This study therefore tests the hypothesis that women will have a stronger response than men to feedback from peers.

## Methods

### Data

Data for this project was drawn from a de-identified archival database of peer and staff affirmations and corrections kept for purposes of tracking unit functioning at two units for men and one unit for women at a single community based correctional TC in the Midwestern United States over a period of several years. Each of the units included eighty beds and was segregated from the others. While the TC was located in a small city, it drew from a catchment area that included a mix of urban, suburban and rural counties. The maximum length of stay in the program was 6 months, but residents could leave sooner depending on clinical progress. All residents were felony offenders who had chosen TC treatment as an alternative to a longer sentence in a correctional facility. The database included 1,162 male residents and 1,032 female residents.

When a resident affirmed or corrected a peer, he or she would do so using a form that included the date, his or her own name, the name of the peer, and the content of the affirmation or correction. A committee of senior residents and staff would then vet the form for legitimacy; for instance, residents were not allowed to correct a peer merely because the peer had recently corrected them. Once it was determined to be legitimate, the affirmation or correction would be read aloud at a time when the entire community was together and would then be entered into a computer database. Because these affirmations and corrections included records of sender, receiver, and the date sent they constitute a longitudinal social network.

The facility also kept records of resident age, race and scores on the Level of Service Inventory-Revised (LSI-R) (36). The LSI-R includes information on substance abuse, education level, previous offenses, employment, financial status, social support, family and marital status, living accommodations, recreational skills, mental health issues, and attitudes toward criminal behavior.

Because this is a de-identified, archival dataset that was originally gathered for clinical purposes, the Ohio State University Office of Responsible Research Practices. ruled that the data did not meet the federal definition of human subjects data.

### Analysis

The dependent variable in the analysis, intended to measure the response to peer feedback, was the number of affirmations sent during a given week. Weekly affirmations were chosen over corrections as a measure of response to peer feedback because qualitative research has shown considerable ambivalence among TC residents about the use of peer corrections (37, 38). This would add random error to the analysis, and therefore any relationship between affirmations/corrections received and affirmations sent should be more easily detected than that between affirmations/corrections received and corrections sent. Testing both affirmations and corrections sent as dependent variables would have been possible but would also have increased the total number of hypothesis tests and therefore the chance of a Type I error.

The predictors of primary interest were the number of affirmations and corrections received during recent weeks of residence. In this study it was expected that women would increase the rate of sending affirmations after receiving either affirmations or corrections more than men would, thus showing a stronger response to feedback. This difference can be measured in two different ways. It is possible that women show a stronger correlation between the affirmations they send and the affirmations or corrections they receive in a given week. However, it is also possible that their response will last for more weeks, tailing off more slowly than that of men. This difference is measured as the total number of lagged weeks before the 95% confidence interval first includes no measurable response. Age, LSI-R and race were entered as demographic control variables; a squared term was entered for age and LSI-R to account for a possible quadratic relationship.

The response variable was a count of affirmations sent. Our objective was to test whether the rate of sending affirmations increases more for women than men under a condition in which the number of affirmations or corrections received has increased. We therefore considered both Poisson and negative binomial models for the errors and landed on the latter due to evidence of over-dispersion in the errors conditional on the Poisson model. We used a log link function. Our data consist of repeated weekly measures of each resident. To account for within-person correlation in the number of affirmations given, we also included an individual-level random effect. We further included TC unit fixed effects and time fixed effects that represent the week of the program that the resident is currently in to adjust for time-in-program confounding of the relationship between the exposure (received interactions) and the outcome (given interactions). The model controlled for age, gender, race and LSI-R of the individuals. In addition to measures of statistical significance, the negative binomial model yields the Incidence Rate Ratio (IRR), the percentage change in the affirmations that residents send per unit of each predictor variable. The IRR provides a valuable tool for understanding the strength of the relationship between the affirmations and corrections that residents receive and the rate of affirmation sending.

The model included a total of 8 lags for received peer interactions of both types, affirmations and corrections. Therefore, the first 8 weeks for which not all lags are available (e.g., week 8 lacks a lag for week 1, week 7 lacks lags for weeks 1 and 2) are excluded from the analysis. Both types of lags are standardized so that one unit is equal to one standard deviation. To test our hypothesis, both sets of lags are interacted with a variable *male* that indicates whether the individual belongs to a male group or the female group (male = 1, female = 0). The lag interaction coefficients represent the difference in the coefficient of each lag for males relative to females, who are represented by the lag main effects. Thus, if an interaction coefficient is negative and statistically significant, it indicates that males have a smaller positive response (or larger negative response) to received interactions than females in terms of the rate of affirmation sent. We defined statistical significance as the case when the 95% coefficient interval did not include zero.

The analysis was completed within the R statistical computing language and environment (39), and the model was constructed and fit with the “brms” addon package to R (40).

## Results

Descriptive statistics are in Table 1. The mean number of corrections received is considerably higher than the mean number of affirmations received; this does not follow the generally assumed guideline that positive reinforcers should outnumber negative reinforcers (41). The mean number of affirmations sent is unsurprisingly virtually identical to the mean number of affirmations received. All of the variables measuring peer feedback have wide ranges, with some residents receiving far more affirmations and corrections than others, and some residents sending far more affirmations. Roughly 31% of residents are Black American.

**Table 1:** Descriptive Statistics for resident activity and demographics.

	**Mean or proportion**	**sd**	**Min**	**Max**
***n*** **=** **2,194**				
Total affirmations received	39.30	60.30	0	306
Total corrections received	60.70	46.53	0	301
Total affirmations sent	39.61	71.92	0	584
Age	29.90	8.76	18	61
LSI-R	25.61	5.74	7	57
Race black american	0.31			
Race other	0.01			

Table 2 gives the results of the statistical analysis, allowing for control of age, race and LSI-R. When female residents receive affirmations they send more affirmations in the same week and for 8 weeks after (Female Response to Affirmations, Lag 0-8). When they receive corrections they send more affirmations in the same week and for 3 weeks after (Female Response to Corrections, Lag 0-3). Overall male residents send more affirmations (Male Affirmation Main Effect). However, when compared to female residents, male residents respond to receiving affirmations more weakly on the initial week and lags 1, 2, and 5-8 (Male Response to Affirmations Lags 0-2, 5-8). They respond to receiving corrections more weakly on lags 2 and 8 (Male Response to Corrections, Lags 2 and 8). There are no lags in which male residents show a statistically significantly stronger response to either affirmations or corrections. Overall, therefore, female residents respond more strongly to feedback whether it comes in the form of affirmations or corrections. Residents who are older send more affirmations on average (b_age). There is no evidence of non-linearity in this relationship, and the analysis found no relationship between LSI-R score or race and the number of affirmations that residents send. Men's unit 1 is more active than men's unit 2 (Unit 1 vs. Unit 2 Males, Affirmation Main Effect).

**Table 2:** Results of multilevel model of response to affirmations and corrections over 8 weekly time lags, by gender.

	**Est**.	**SE**	**IRR**	**IRR 95% CI**		**Significance (*p* ≤ 0.05)**
				**Lower**	**Upper**	
b_Intercept	-1.659	0.115	0.192	0.153	0.241	*
Female response to affirmations	0.669	0.030	1.952	1.844	2.064	*
Female response to affirmations, lag 1	0.332	0.029	1.395	1.319	1.473	*
Female response to affirmations, lag 2	0.245	0.028	1.278	1.210	1.349	*
Female response to affirmations, lag 3	0.182	0.028	1.201	1.138	1.266	*
Female response to affirmations, lag 5	0.165	0.029	1.179	1.115	1.248	*
Female response to affirmations, lag 5	0.215	0.028	1.241	1.173	1.312	*
Female response to affirmations, lag 6	0.184	0.027	1.202	1.141	1.269	*
Female response to affirmations, lag 7	0.160	0.028	1.173	1.109	1.235	*
Female response to affirmations, lag 8	0.175	0.026	1.191	1.130	1.256	*
Female response to corrections	0.166	0.030	1.181	1.113	1.247	*
Female response to corrections, lag 1	0.064	0.030	1.066	1.008	1.128	*
Female response to corrections, lag 2	0.087	0.029	1.092	1.030	1.156	*
Female response to corrections, lag 3	0.059	0.028	1.062	1.006	1.122	*
Female response to corrections, lag 4	0.024	0.029	1.025	0.967	1.084	
Female response to corrections, lag 5	0.058	0.030	1.060	0.999	1.123	
Female response to corrections, lag 6	-0.010	0.028	0.990	0.939	1.045	
Female response to corrections, lag 7	0.015	0.028	1.016	0.962	1.073	
Female response to corrections, lag 8	0.064	0.029	1.067	1.008	1.130	*
Male affirmation main effect	1.790	0.117	6.030	4.779	7.516	*
b_age	0.130	0.057	1.141	1.015	1.279	*
b_IageE2	-0.041	0.044	0.960	0.884	1.051	
b_lsir	0.033	0.050	1.034	0.935	1.146	
b_IlsirE2	0.012	0.031	1.012	0.954	1.075	
b_race.AfAmer	0.088	0.112	1.099	0.883	1.372	
b_race.Other	-0.854	0.709	0.542	0.111	1.601	
Unit 1 males compared to Unit 2 males, affirmation main effect	−2.89019	0.14013	0.056115	0.042149	0.0735	*
Male response to affirmations	-0.11417	0.037693	0.892739	0.830224	0.962475	*
Male response to affirmations, lag 1	-0.13559	0.03621	0.873773	0.813823	0.934824	*
Male response to affirmations, lag 2	-0.08852	0.036683	0.915899	0.853896	0.98438	*
Male response to affirmations, lag 3	-0.06789	0.037302	0.935016	0.870935	1.006475	
Male response to affirmations, lag 4	-0.03144	0.037708	0.969733	0.898725	1.040831	
Male response to affirmations, lag 5	-0.1426	0.037075	0.867694	0.805087	0.930775	*
Male response to affirmations, lag 6	-0.0776	0.034951	0.925896	0.861713	0.990202	*
Male response to affirmations, lag 7	-0.07402	0.036406	0.929269	0.862949	0.999936	*
Male response to affirmations, lag 8	-0.1015	0.03509	0.90404	0.843736	0.966583	*
Male response to corrections	-0.03183	0.044946	0.969652	0.890574	1.058374	
Male response to corrections, lag 1	-0.03276	0.046417	0.968811	0.880963	1.056039	
Male response to corrections, lag 2	-0.08642	0.042322	0.918027	0.843666	0.996153	*
Male response to corrections, lag 3	0.047995	0.042648	1.05012	0.96291	1.141858	
Male response to corrections, lag 4	-0.02749	0.042329	0.973752	0.895851	1.053951	
Male response to corrections, lag 5	0.013187	0.04228	1.01418	0.930557	1.101034	
Male response to corrections, lag 6	-0.00065	0.038061	1.000073	0.932383	1.076677	
Male response to corrections, lag 7	0.039281	0.040984	1.040936	0.961073	1.130501	
Male response to corrections, lag 8	-0.11036	0.041037	0.896266	0.825592	0.967112	*
sd_id__Intercept	1.715259	0.052019				*
shape	0.458854	0.009793				*

*The asterisks mark when a particular parameter is a statistically significant predictor*.

**Figure 1:**
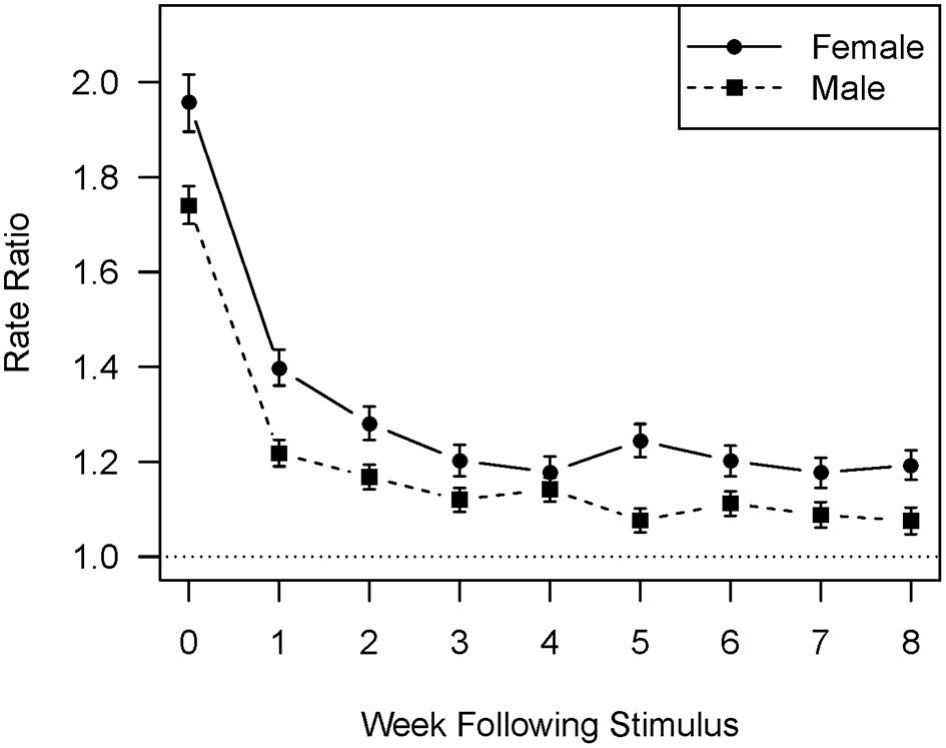
Rate ratios comparing the rate of affirmation giving with and without the stimulus of one standard deviation increase in received affirmations by week since the stimulus and by gender.

**Figure 2:**
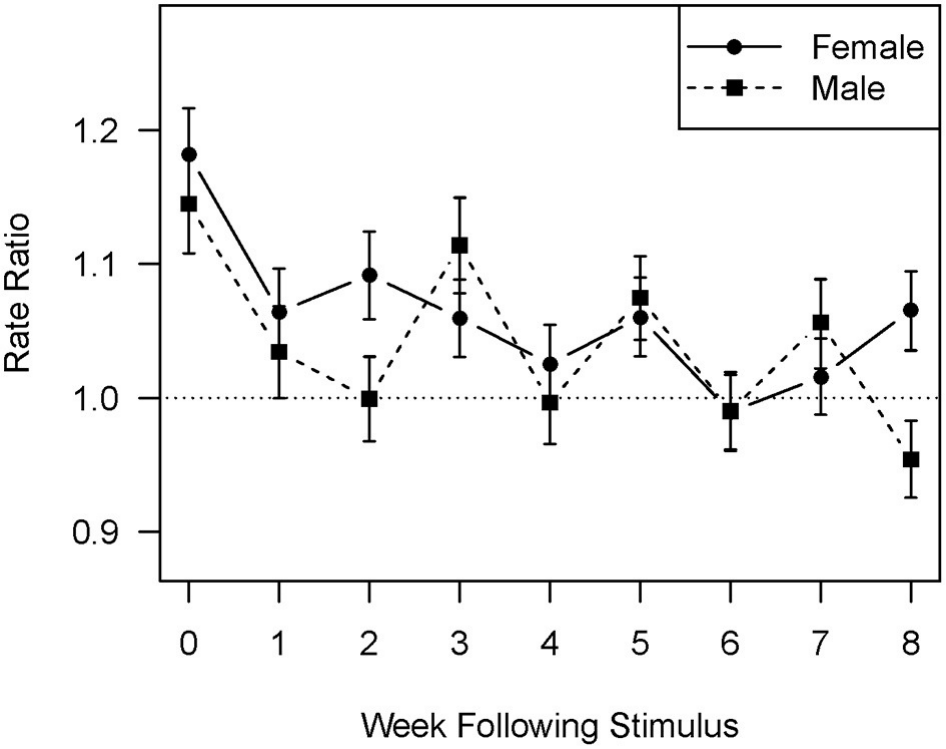
Rate ratios comparing the rate of affirmation giving with and without the stimulus of one standard deviation increase in received corrections by week since the stimulus and by gender.

Figures 1, 2 show the difference in response between men and women to affirmations and corrections, respectively, using a one standard error confidence interval. In the case of affirmations received (Figure 1) the risk ratio for female residents is consistently higher than that for male residents, although the difference slips below statistical significance during the 3 and 4 week lags. For both men and women the correlation shows a smooth exponential decline. In the case of corrections received Figure 2 makes it clear that two factors combine to limit the number of lags on which the difference between men and women is statistically significant. The first is that the correlation between corrections received and affirmations sent is substantially weaker for both genders than that between affirmations received and those sent. The second is that the response of the male residents shows a two lag periodicity, increasing on lags 3, 5, and 7 when compared to the previous lag. The female response appears to have a somewhat weaker three lag periodicity, with increases on lags 2, 5, and 8. This combination leads to statistically significant differences on lags 2 and 8 only, in both cases favoring women.

## Discussion

This study tested the hypothesis that female residents of TCs would respond more strongly to changes in exposure to affirmations or corrections received from peers in terms of their own rate of giving affirmations to the community. The results supported our hypothesis, suggesting that women in the women's TC unit responded more strongly in the positive direction to increases in received affirmations and that the response lasted a larger number of weeks. The same was true to a lesser degree of received corrections. Residents showed a weaker response to corrections than to affirmations, a result that is consistent with the literature on positive vs. negative reinforcement (41). The two male units also showed somewhat different responses, consistent with previous literature on the variability of unit atmosphere in TCs (42).

While these findings are encouraging for the treatment of women in TCs, several caveats must be noted. First, the external validity of this analysis is limited due to the small number of units studied. The men's units themselves showed some variation in the number of affirmations sent, suggesting that differing unit cultures could be an alternative explanation for the differences found between men and women. With a sample of only three units we cannot rule this out. Moreover, peer affirmations and corrections are only one form that feedback takes in TCs, and it is possible that these findings would be different if we looked at other forms of feedback, for instance feedback in therapy groups. Finally, the women in this TC were segregated from men. It is possible that both men and women in facilities that do not segregate genders would show different response patterns to peers either because the stimulus came from a person of another gender (32) or because people of another gender are present, thus changing the overall social dynamic (16). All of that having been said, these findings are consistent with previous experimental studies of comparative gender responsiveness to feedback, which find women to be more responsive than men (33, 34, 43). This consistency indicates that they are likely not artifactual.

With those limitations in mind, these results have several implications for researchers and clinicians involved in substance abuse treatment. The difference in response to affirmations and corrections between the male and female units occur on multiple lags, and the IRR values suggest that these differences are not trivial. For instance, in week five male residents are roughly 13% less likely to send affirmations per standard deviation of the number of affirmations they received in week 0. A likely explanation, as found in previous experimental work on gender and feedback, is that women in TCs perceive feedback as being of more value (34).

These results suggest that women may adapt more easily to the TC system of mutual feedback. It is likely that their reaction to feedback allows them to gain more from the TC system of social learning (1, 44). It is also possible that it indicates and/or fosters a stronger identification with the community, an interpretation that would be consistent with findings that women value relationships more strongly than men (30, 32). Such social identification is increasingly seen as a factor in successful treatment outcomes (45, 46). When taken together with laboratory studies that find that men are less responsive to feedback (33, 34, 43) it raises the possibility that resident training in exchanging feedback in TCs could be useful, and particularly for male residents.

This examination of gender differences in one aspect of TC treatment makes it clear that the question is not so much whether a given treatment program is appropriate for women who abuse substances a (19, 47, 48) as which specific aspects of a program are of more or less benefit for men or women, and by extension other groups. It has been argued on theoretical grounds that the TC system of corrections is inappropriate for women (47). This analysis finds no evidence for that proposition. While TC residents on the whole respond more positively to affirmations than to corrections, women do not respond less positively to corrections than do men, and to some extent respond in a somewhat more pro-social manner.

There was variation between the two men's units in the mean number of peer affirmations sent. The use of the community as therapeutic agent is central to TC practice (1, 2), and this level of variability in amount of positive feedback received from peers suggests that attaining fidelity in peer interaction is likely to be a challenge. This finding is consistent with variability between units found in other recent work on TCs (42) and, like the difference in response between men and women, raises the possibility that training for TC residents on how to exchange feedback with peers might be of value.

Consistent with earlier analysis (35) and behavioral treatment literature (41), this one found that residents responded more strongly to affirmations than to corrections; this was true for both the effect size during the initial and subsequent weeks and the length of time over which the effect was observable. This analysis adds the important nuance that response to corrections appears to be more complex than response to affirmations, with periodicity being visually evident in Figure 2. Such periodicity is consistent with evidence that attempts to constrain antisocial behavior can create non-linearities that lead to complex periodicities in time series (49, 50) and would be expected to add to the complexity of the clinical task of monitoring unit functioning. While there is no apparent periodic component in resident response to affirmations, it is possible that the narrowing of the difference between men and women on the third and fourth lags represents a much weaker periodic element in the time series that is not clearly visible in the graphs.

While the difference in resident response to affirmations and corrections intuitively suggests that rewards change behavior more effectively than punishments, it is important to distinguish between response to affirmations or corrections and learning from affirmations or corrections. This analysis does not preclude the possibility of long-term learning from corrections (1, 2) or the possibility that a willingness to correct peers may be part of a process of personal growth, increasing social identification with the community and role modeling that is itself important in recovery (1, 2, 45, 46, 51, 52).

## Conclusion

Consistent with laboratory research, this study found that female residents of TCs have a stronger response to peer feedback than male residents of TCs, and that the difference in response to affirmations in particular is statistically detectable over 8 weeks. It also found a weaker overall response to corrections and a visually apparent periodicity in response to corrections. As TC clinicians adapt their programs to the needs of women, these findings support the continued use of peer feedback, while demonstrating that residents react more immediately and in a more straightforward manner to affirmations.

This analysis also shows that theory and empirical findings from outside the clinical literature specific to substance abuse treatment can be of use in understanding these complex programs. Because TCs depend on the community of recovering peers as the primary method of clinical treatment, empirical studies of interpersonal interactions in other settings are particularly likely to be of relevance.

## Data Availability Statement

The data analyzed in this study is subject to the following licenses/restrictions: The dataset is currently de-identified. We can share it with individuals who wish to replicate the study. Requests to access these datasets should be directed to warren.193@osu.edu.

## Ethics Statement

The studies involving human participants were reviewed and approved by The Ohio State University Office of Responsible Research Practices. Written informed consent for participation was not required for this study in accordance with the national legislation and the institutional requirements.

## Author Contributions

ND did the data analysis, wrote the methodology section, and contributed to writing the results. FD and KW did editorial work on the entire manuscript and wrote the introduction. KW wrote the results, discussion, and conclusion. All authors contributed to conceptualizing this manuscript.

## Conflict of Interest

The authors declare that the research was conducted in the absence of any commercial or financial relationships that could be construed as a potential conflict of interest.
